# Pexidartinib and Nintedanib Combination Therapy Targets Macrophage Polarization to Reverse Pulmonary Fibrosis: A Preclinical Study

**DOI:** 10.3390/ijms26157570

**Published:** 2025-08-05

**Authors:** Ji-Hee Kim, Jae-Kyung Nam, Min-Sik Park, Seungyoul Seo, Hyung Chul Ryu, Hae-June Lee, Jeeyong Lee, Yoon-Jin Lee

**Affiliations:** 1 Division of Radiation Biomedical Research, Korea Institute of Radiological and Medical Sciences, Seoul 01812, Republic of Korea; wlgml9054@kirams.re.kr (J.-H.K.); hjl22@jejunu.ac.kr (H.-J.L.);; 2 Agathonbio Co., Ltd., Hwaseong-si 18487, Gyeonggi-do, Republic of Korea; syseo@agathonbio.com; 3 Independent Researcher, Hwaseong-si 18487, Gyeonggi-do, Republic of Korea; rosen-ryu@astrogen.co.kr; 4 College of Veterinary Medicine, Jeju National University, Jeju 63243, Republic of Korea

**Keywords:** nintedanib, pexidartinib, PLX3397, combination therapy, idiopathic pulmonary fibrosis

## Abstract

Idiopathic pulmonary fibrosis (IPF) is a chronic, progressive interstitial lung disease with limited therapeutic options and increasing global incidence, with a median survival of only 2–5 years. The clinical utility of macrophage polarization to regulate the progression of pulmonary fibrosis remains understudied. This study determined the efficacy of nintedanib and pexidartinib (PLX3397) combination therapy for treating IPF. Combination treatment effectively inhibited the progression of radiation-induced pulmonary fibrosis (RIPF) and prolonged survival in bleomycin-treated mice. Micro-CT analysis revealed a significant tissue repair efficacy. The therapy significantly normalized the abnormal vascular structure observed during RIPF and bleomycin-induced pulmonary fibrosis progression and was accompanied by a decrease in the M2 population. Polarized M1 macrophages enhanced normalized tube formation of irradiated endothelial cells (ECs) in vitro; M2 macrophages increased adhesion in irradiated ECs and abnormal tube formation. Single-cell RNA sequencing data from patients with IPF further supports colony stimulating factor (CSF) 1 upregulation in macrophages and downregulation of capillary EC markers. This study highlights a promising combination strategy to overcome the therapeutic limitations of monotherapy with nintedanib for the treatment of IPF.

## 1. Introduction

Idiopathic pulmonary fibrosis (IPF) is a chronic, progressive interstitial lung disease characterized by the development of fibrosis of unknown cause. The incidence and prevalence of IPF are increasing globally, and this increase was evident before the onset of the COVID-19 pandemic. The median survival of patients with IPF ranges from 2 to 5 years after diagnosis [1,2,3].

The approval of two antifibrotic therapeutic agents, pirfenidone and nintedanib, marked a significant milestone in the treatment of IPF. Nintedanib is a tyrosine kinase inhibitor composed of small molecules that selectively target fibroblast growth factor (FGF), platelet-derived growth factor (PDGF), and vascular endothelial growth factor receptor (VEGF) [4,5]. Pirfenidone inhibits the production of transforming growth factor-stimulated collagen, thereby reducing fibroblast proliferation [6,7]. Despite the effectiveness of antifibrotic therapeutic agents in slowing down the decline in lung function, disease progression does not halt; therefore, the development of therapeutic drugs for the treatment of IPF remains crucial [8]. Further, despite efforts in the development of idiopathic fibrosis inhibitors, an effective drug target has not yet been identified. Furthermore, as drug development primarily focuses on inhibiting collagen synthesis, it becomes challenging to address the progression of fibrosis, which is influenced by diverse factors. Since the pathogenesis of IPF is associated with multiple factors, it emerges as an optimal candidate for combined therapeutic approaches [9]. However, unexpected drug–drug interactions (pirfenidone with the antioxidant N-acetylcysteine) can lead to poor outcomes [10]. Vancheri et al. [11] provided valuable insights into the feasibility of combined treatment (nintedanib and pirfenidone) for patients with IPF [12].

Immune dysregulation is a key driver in the development of pulmonary fibrosis (PF) [13]. Notably, macrophages have a remarkable ability to display plasticity and acquire phenotypes that can either promote or ameliorate fibroproliferative responses to lung injury, particularly in fibrotic areas that resemble M2-like macrophages. Macrophages are highly plastic immune cells that can polarize into distinct phenotypes. M1 macrophages, induced by IFN-γ, are pro-inflammatory, whereas M2 macrophages, activated by IL-4 and IL-13, promote tissue fibrosis. In chronic inflammation, sustained M2 polarization contributes to fibrosis by enhancing transforming growth factor-β (TGF-β) production, activating fibroblasts, and promoting excessive extracellular matrix (ECM) deposition. A predominance of M2 macrophages has been observed in idiopathic pulmonary fibrosis (IPF) and other fibrotic diseases, as well as in tumor microenvironments. The aberrant accumulation of M2 macrophages in fibrotic tissues has thus emerged as a critical factor in the pathogenesis of fibrosis and a promising therapeutic target [14,15,16]. Macrophage polarization is regulated by various factors, among which colony-stimulating factor 1 receptor (CSF1R) signaling plays a crucial role in determining the M1/M2 macrophage phenotype [17].

CSF1R is a cell surface receptor involved in the regulation of the immune system, particularly in the development and function of macrophages. Thus, anti-CSF1 antibodies or PLX3397 (a small-molecule inhibitor of M-CSF1R kinase signaling) decrease the severity of PF [17,18]. Furthermore, targeting macrophage diversity causes tissue fibrosis and regeneration [19].

Vascular abnormalities and endothelial cell (EC) dysregulation are hallmarks of IPF. Dense capillary structures form during the initial phase of fibrosis, whereas during fibrosis, pulmonary vasculature structures are absent [20]. Previously, we demonstrated that endothelial-to-mesenchymal transition induces vascular changes and tissue hypoxia, accelerating fibrosis progression during the development of radiation-induced PF (RIPF) [21]. While it is known that one of the targets of nintedanib, VEGFR, exerts antifibrotic effects, its specific impact on pulmonary ECs, which constitute approximately 30% of the pulmonary cells and are a major component of the alveoli, remains unclear [22]. The progressive vascular pathology of ECs can lead to tissue destruction. Nonselective phosphodiesterase-4 (PDE4) inhibitors, which protect against cardiac dysfunction, limit fibrosis, and vascular remodeling in bleomycin-induced PF (BIPF) mouse models. Moreover, in a recent phase II trial, a preferential PDE4B inhibitor was proven effective in the treatment of IPF [23]. Recently, the promotion of endothelial repair has been explored as a potential IPF treatment strategy [24]. M2 polarization of macrophages regulates the fibrotic response in tissues [17]. Further, M-CSF/M-CSF1R signaling in monocyte-derived alveolar macrophages is crucial in PF [17].

Thus, we hypothesized that modulating macrophage polarization, with the use of PLX3397, to regenerate fibrotic tissue would enhance the therapeutic effects of nintedanib and inhibit fibroblast proliferation. Therefore, in this study, we aimed to determine the efficacy of nintedanib and PLX3397 combination therapy for the treatment of IPF.

## 2. Results

### 2.1. Enhanced Efficacy of Nintedanib in RIPF Through Combined Treatment with PLX3397

**Figure 1:**
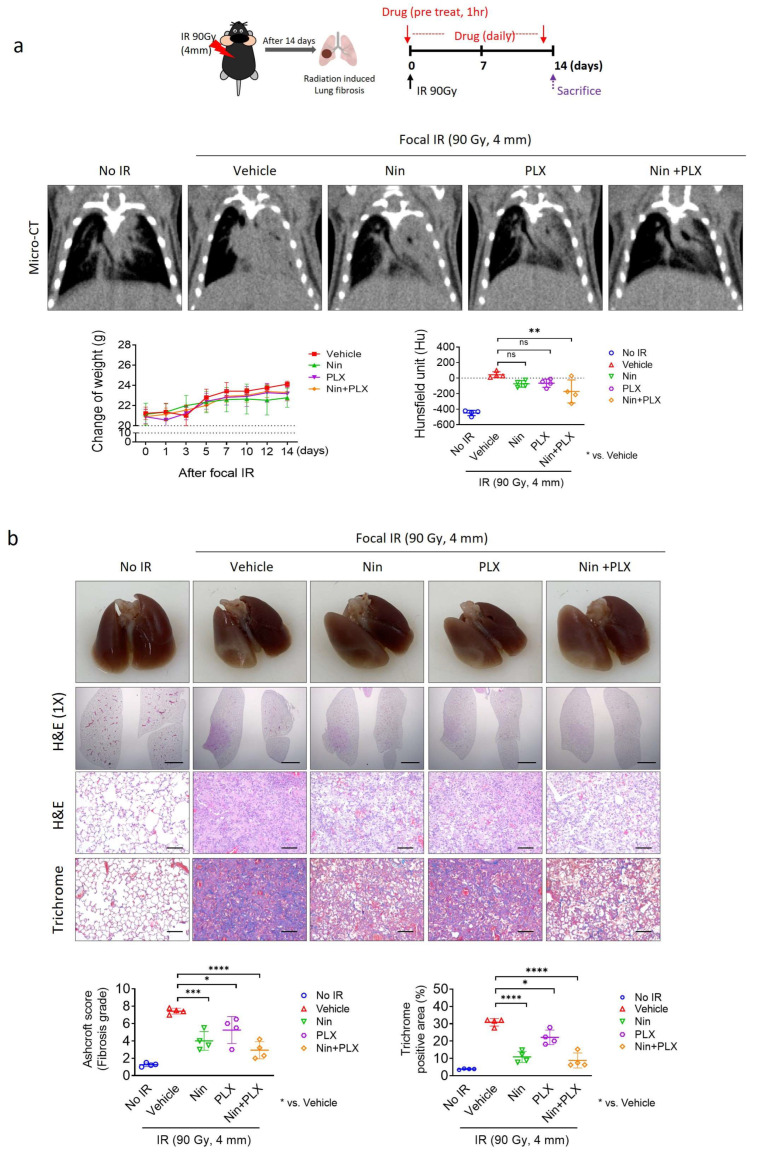
The effects of the combined PLX3397 and nintedanib therapy on RIPF progression: Micro-CT imaging and histopathological analysis. Radiation exposure of 90 Gy was administered using a 4 mm-diameter field to mice aged 7-to-8 weeks old. Following a 14-day period post-irradiation, mice were orally administered Nin (Nintedanib, 60 mg/kg), PLX (PLX3397, 60 mg/kg), or a combination of Nin and PLX (Nintedanib and PLX3397, 60 mg/kg each), once daily for 2 weeks (*n* = four animals per group). (**a**) Micro-CT images depicting the lungs of mice in the fibrosis phase, 2 weeks post-irradiation. Quantification of body weight (left graph) and lung density, Hounsfield unit (HU) (right graph). (**b**) Following a 14-day post-irradiation period, the mice were orally administered Nin (60 mg/kg), PLX (60 mg/kg), or a combination of Nin and PLX (60 mg/kg each) once daily for 2 weeks. H&E and Masson’s trichrome staining were conducted on the lungs of mice 2 weeks post-irradiation. Scoring of fibrosis grade and quantification of collagen deposition per field (magnification: 200×). (scale bar = 2.5 µm). For the Ashcroft graph, the error bars indicate the SD. For other graphs, error bars indicate the SEM images. * *p* < 0.05, ** *p* < 0.01, *** *p* < 0.001, **** *p* < 0.0001, and ns: not significant (one-way ANOVA for multiple comparison).

To enhance the efficacy of nintedanib, which is currently in clinical use, we aimed to evaluate the effects of the combined treatment of nintedanib and PLX3397 in a mouse model of RIPF. At 14 days after irradiation, PF was observed using micro-CT images, with a significant alteration in the gray scale (Figure 1a). No change in body weight was observed during the 14-day period of treatment administration (single and combination therapy) following focal irradiation, compared to the vehicle-treated group (Figure 1a; Bottom). Analysis of the micro-CT images revealed that nintedanib and PLX3397 exhibited inhibitory effects on RIPF. The inhibitory effect of the combination treatment on RIPF was significantly higher than that in the nintedanib-treated group (Figure 1a). As shown by hematoxylin and eosin (H&E) and trichrome staining, irradiation induced marked fibrotic alterations characterized by the loss of normal alveolar architecture and cellularity, depletion of nucleated cells, inflammatory cell infiltration, and extensive collagen deposition. Both nintedanib and PLX3397 monotherapies inhibited RIPF, as evidenced by reduced blue areas in trichrome-stained sections and partial preservation of alveolar architecture. Notably, the combined treatment resulted in a greater reduction in fibrotic lesions, with markedly restored alveolar structure and decreased collagen deposition (Figure 1b). These results suggest that combined treatment with nintedanib and PLX3397 inhibits RIPF to a greater extent than either treatment alone.

### 2.2. Effect of the PLX3397 and Nintedanib Combination Therapy on Macrophage Polarization During RIPF Development

**Figure 2:**
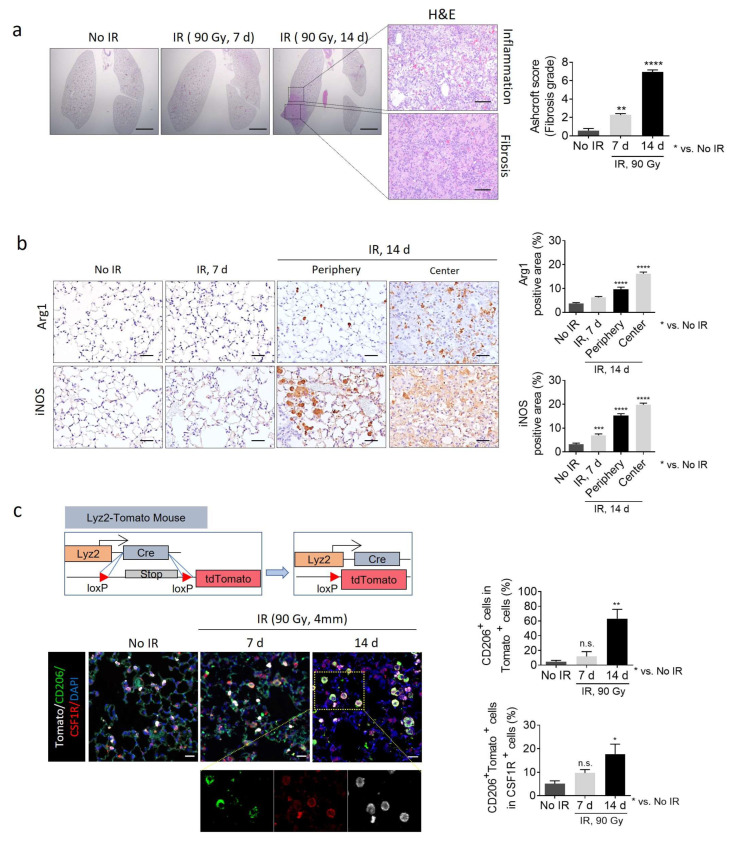
Temporal changes in lung tissue histology and macrophage polarization following radiation exposure. (**a**) C57BL/6 mice were exposed to 90 Gy of radiation directed at the left main bronchus using a 4 mm diameter field. Lung tissues were obtained at 0-, 7-, and 14 days post-irradiation. Representative images of the 10× lungs; hematoxylin and eosin (H&E) in the lung tissues from C57BL/6 mice with or without irradiation. Lung tissues from C57BL/6 mice at 0-, 7-, and 14 days post-irradiation reveal inflammation and fibrosis areas after 14 days. A significantly elevated inflammatory response was observed in the irradiated group relative to the non-irradiated group. (**b**) Immunohistochemistry staining for Arg1 and iNOS was performed on lung tissues obtained from C57BL/6 mice at 7- and 14 days post-irradiation or non-irradiation. The graphs show the areas positive for Arg1 and iNOS. The average of five fields of Arg1, iNOS positive area (%) was quantified (magnification: 200×). (**c**) Lyz2-Cre; tdTomato mice were generated by crossing Lyz2-Cre and CAG-tdTomato mice. Immunofluorescence staining of DAPI (blue), CD206 (green), CSF1R (red), and tdTomato (white) was conducted on lung tissues obtained from Lyz2-Cre and tdTomato mice obtained at 7-, 14-, and 21 days post-irradiation or non-irradiation. The graphs indicate CD206^+^ cells (%) in tdTomato+ cells and CD206^+^tdTomato^+^ cells in CSF1R^+^ cells. The scale bar for the H&E image was set to 50 µm, and immunohistochemistry and immunofluorescence images were set to 20 µm. The average of five fields of Arg1, iNOS density was quantified (magnification: 200×). For the Ashcroft graph, error bars indicate SD. For other graphs, error bars indicate SEM. * *p* < 0.05, ** *p* < 0.01, *** *p* < 0.001, **** *p* < 0.0001, and ns: not significant (one-way ANOVA for multiple comparisons).

Targeting CSF1R, which is involved in fibrotic M2 macrophage polarization, delays processes such as PF, RIPF, and IPF [17,18]. However, the clinical significance of the regulation of macrophage polarization remains poorly understood. As shown in Figure 2a, during the development of RIPF, a severe fibrotic area was observed in the irradiated center 14 days after irradiation, and inflammatory regions were observed in the periphery of this fibrotic area. iNOS-positive M1 macrophages accumulate at the inflammatory periphery of the fibrotic region, occurring approximately 14 days after irradiation. Conversely, in the center area of the fibrotic tissue, there is a prominent accumulation of arginase-1 (Arg1)-positive M2 macrophages. Accordingly, Arg1 and iNOS expression were quantified separately in the center and periphery at day 14, when this spatial distinction is evident (Figure 2b). We developed mice capable of tracing macrophages by crossing monocyte-specific Cre (Lyz2-CRE) mice with Loxp-td tomato mice (Figure 2c). Thus, in the irradiated fibrotic lung tissue 14 days after irradiation, Cd206^+^ cells in Tomato^+^ cells comprised more than 60%. Furthermore, most of the Cd206^+^Tomato^+^ cells exhibited high expression of CSF1R, indicating that most M2 macrophages expressing CSF1R during RIPF were monocyte-derived (Figure 2c).

**Figure 3:**
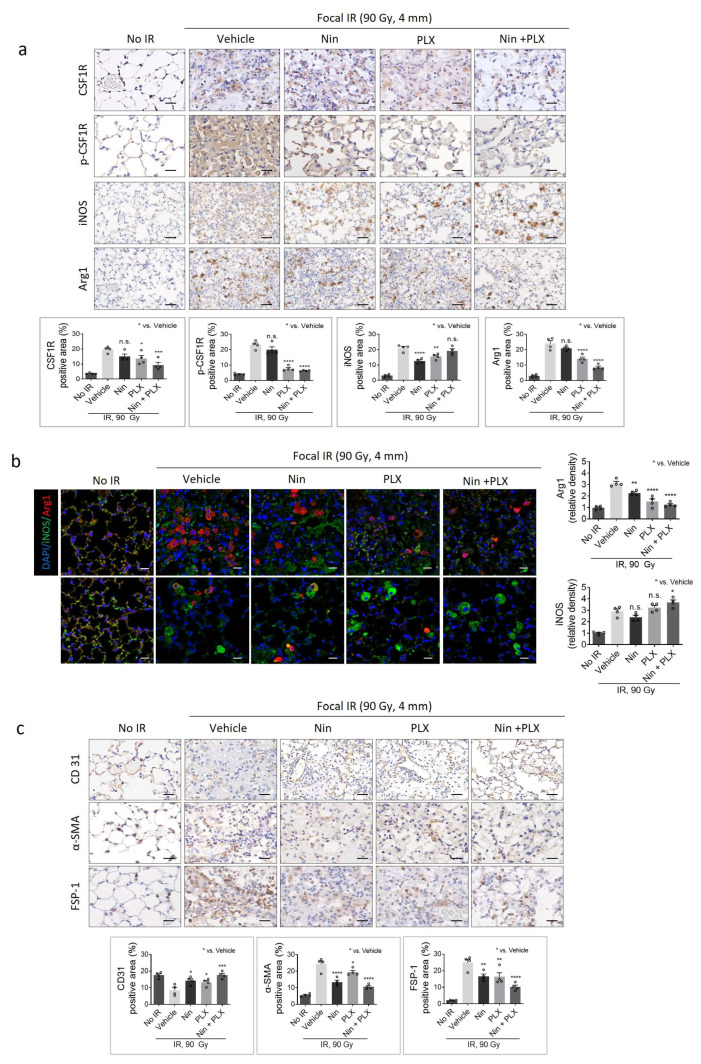
Effects of PLX3397 and nintedanib combined therapy on macrophage polarization during RIPF. C57BL/6 mice were irradiated in the left main bronchus with 90 Gy using a 4 mm diameter field. Following a 14-day period post-irradiation, mice were orally administered Nin (60 mg/kg), PLX (60 mg/kg), and a combination of Nin and PLX (60 mg/kg each), once daily for 2 weeks (*n* = 4 animals per group). (**a**) Immunohistochemistry staining of CSF1R, p-CSF1R, Arg1, and iNOS in the lung tissue of C57BL/6 mice 2 weeks after irradiation or non-irradiation; CSF1R and p-CSF1R scale bar = 10 µm, Arg1 and iNOS scale bar = 20 µm. The graphs show the positive area of CSF1R, p-CSF1R, Arg1, and iNOS. The average of five fields of CSF1R, p-CSF1R, iNOS, and Arg1 positive area (%) was quantified (magnification: 200×). (**b**) Immunofluorescence staining of DAPI (blue), Arg1 (red) and iNOS (green) colocalization in lung tissues (scale bar = 20 µm, magnification: 200×). (**c**) Immunohistochemistry staining of CD31, α-SMA, and FSP-1 in the lung tissue of C57BL/6 mice 2 weeks after irradiation or non-irradiation. The graphs show the positive area (%) of CD31, α-SMA, and FSP-1. The average of five fields of all images was quantified by magnification 200×. For all graphs, error bars indicate SEM. Each circle in the bar graph represents the mean value of an individual. * *p* < 0.05, ** *p* < 0.01, *** *p* < 0.001, **** *p* < 0.0001 and ns: not significant (one-way ANOVA for multiple comparisons).

As shown in Figure 3a, immunohistochemistry (IHC) analysis showed that CSF1R and phospho-CSF1R were overexpressed in the irradiated lung tissue of the vehicle group, which was significantly inhibited by nintedanib and PLX3397 combination treatment. Both nintedanib and PlX3397 reduced the population of Arg1-positive macrophages in irradiation-induced fibrotic regions. Furthermore, the combined treatment notably reduced the M2 macrophage population compared to each treatment alone while increasing the population of iNOS-positive M1 macrophages around the fibrotic region (Figure 3a,b).

In accordance with the fibrotic severity (Figure 1b), the expression of α-SMA and FSP-1, markers of myofibroblast activation, increased as RIPF progressed, a phenomenon significantly attenuated by the concurrent administration of nintedanib and PLX3397 (Figure 3c). Additionally, severe damage to the alveolar structure was evident in RIPF tissue, accompanied by a significant reduction in CD31-positive vessels, particularly capillary ECs. However, treatment with nintedanib or PLX3397 alone effectively preserved capillary vessel density. Furthermore, concurrent administration of nintedanib and PLX3397 demonstrated robust preservation of both capillary vessel density and alveolar structure.

### 2.3. Modulation of Macrophage Polarization-Mediated Vascular Effects in RIPF

**Figure 4:**
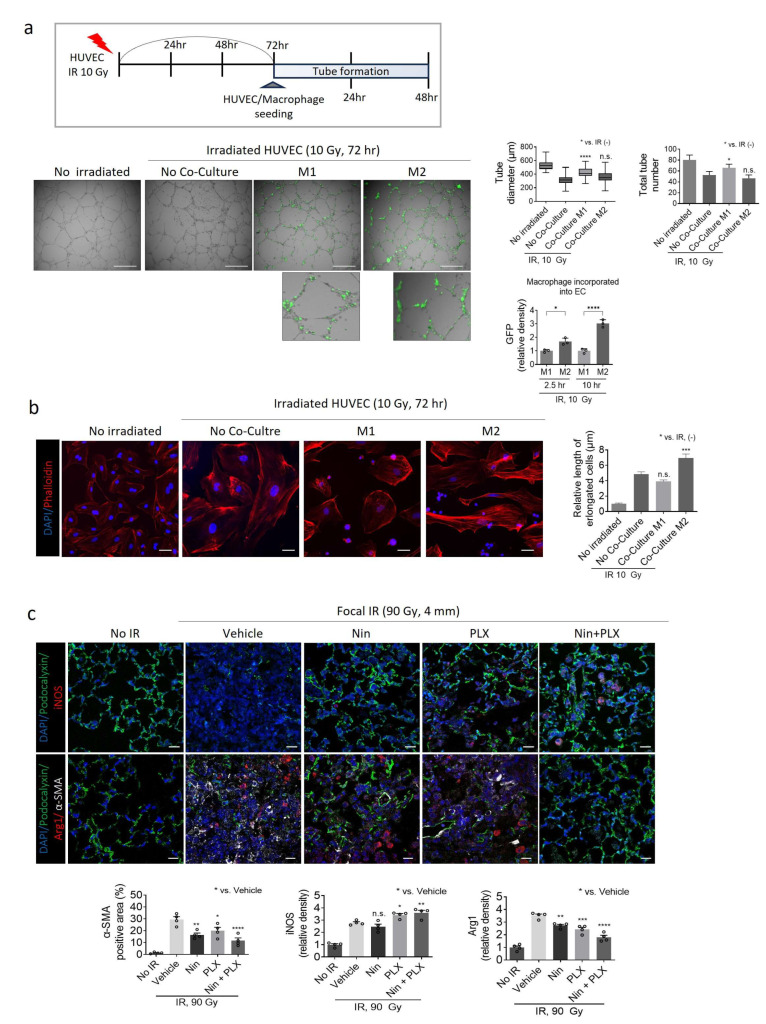
Comparative analysis of macrophage-associated responses on EC in vitro and lung tissue of RIPF. (**a**) HUVEC tube formation in the M1 and M2 macrophages after irradiation. Human monocytes, differentiated by treatment with GM-CSF and M-CSF every other day for 7 days, were placed on the HUVECs. Tube analyzed at 10 h after seeding. Green fluorescence indicates M1 and M2 macrophages. The graphs show the average tube diameter, total tube number, and relative GFP density (scale bar = 650 µm). (**b**) HUVEC and THP-1 co-cultured after HUVEC irradiation at 10 Gy. Immunofluorescence of phalloidin (red) and DAPI (blue) after a 72 h co-culture with HUVEC with M1 macrophage and M2 macrophage (scale bar = 50 µm). The graph shows that the average length of phalloidin filaments per cell (average of at least 30 cells) was quantified in five. (**c**) Immunofluorescence staining of Podocalyxin (green), Arg1 (red), iNOS (red), DAPI (blue) and α-SMA (white) and in the lung tissue of C57BL/6 mice 4 weeks after bleomycin injection (scale bar = 20 µm). The graph shows the percentage of α-SMA area and relative density of iNOS and Arg1. For graphs, tube formation error bars indicate SD, and the others are SEM. Each circle in the bar graph represents the mean value of an individual. * *p* < 0.05, ** *p* < 0.01, *** *p* < 0.001, **** *p* < 0.0001 and ns: not significant (one-way ANOVA for multiple comparisons).

Recent studies have reported the inhibition of PF through the modulation of macrophage M2 polarization [25]. However, the direct pathological changes resulting from the inhibition of M2 polarization during PF development remain unclear. Therefore, we investigated whether the inhibition of M2 polarization by co-current treatment with nintedanib and PLX3397 during RIPF directly affected vascularization. Consistent with our previous findings, pulmonary vascular ECs undergo endothelial-to-mesenchymal transition, transforming into a fibrotic cell type [21,26]. Human primary ECs exhibited abnormal and irregular tube formation 72 h after radiation treatment compared to untreated ECs (Figure 4a).

Subsequently, we examined whether M1 and M2 macrophages exhibited differential effects on tube formation in irradiated ECs (Figure 4a). M1 and M2 macrophages were differentiated from human monocytes following treatment with GM-CSF and M-CSF, respectively. M2 macrophages showed significantly increased abnormal tube formation upon incorporation into ECs, displaying elongated phenotypic changes with a highly increased density of GFP fluorescence, indicative of cytoskeletal remodeling and a fibrotic transition. Conversely, M1 macrophages did not exacerbate abnormal tube formation in irradiated ECs and did not incorporate well into irradiated tube ECs (Figure 4a).

In a supportive manner, the fibrotic phenotype of the irradiated ECs transitioned to a non-fibrotic state with reduced cytoskeletal filaments when co-cultured with M1 macrophages. However, when co-cultured with M2 macrophages, the ECs exhibited a more elongated phenotype (Figure 4b).

The development of RIPF significantly reduces podocalyxin-positive vessel intensity, an EC marker, in vehicle-treated fibrotic tissues, along with severe destruction of alveolar structures and noticeable abnormalities in the increased SMA^+^ area (Figure 4c). These effects were mitigated upon treatment with nintedanib and PLX3397. Co-treatment with nintedanib and PLX3397 notably increased the population of iNOS^+^ M1 macrophages while reducing Arg1^+^ M2 macrophages, preserved the sharpness of the EC lining compared to vehicle-treated fibrotic tissue, and effectively protected the alveolar architecture (Figure 4c).

Based on these findings, we propose that the concurrent administration of nintedanib and PLX3397 targets the polarization of M2 macrophages, thereby inhibiting M2 macrophage-mediated abnormal vascularization, consequently attenuating the fibrotic process.

### 2.4. Synergistic Effects of Nintedanib and PLX3397 in Restoring BIPF and Prolonging Survival

Bleomycin, initially introduced as an anticancer drug, is now widely used in experimental models of pulmonary fibrosis due to its well-characterized lung toxicity. Its ability to induce epithelial cell injury through oxidative stress and DNA damage initiates a cascade of fibrotic responses, including the activation of TGF-β signaling [16,27].

**Figure 5:**
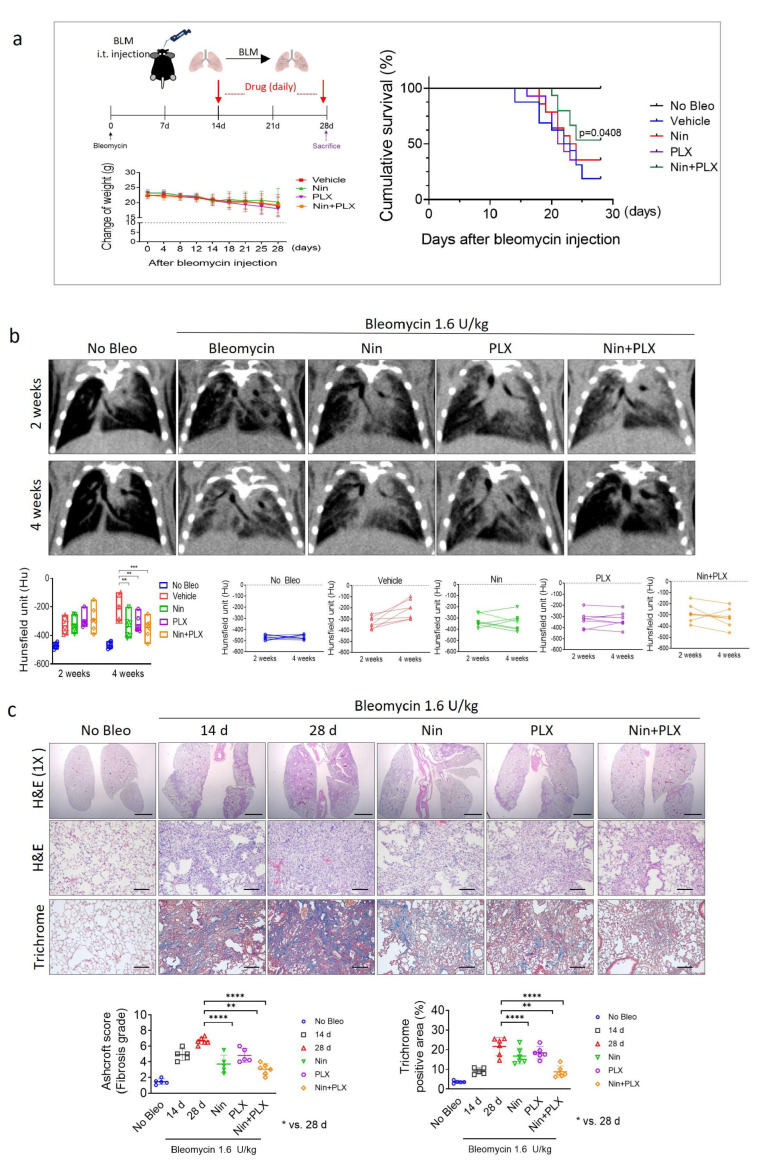
Synergistic effects of nintedanib and PLX3397 in IPF, and prolonging survival in bleomycin-treated mice. Mice were injected intratracheally with bleomycin (1.6 U/kg) in 60 μL PBS. Mice were orally administered 60 mg/kg of Nin, PLX, or a combination of Nin and PLX 14 days after bleomycin administration. (**a**) Cumulative survival and change in weight analysis measured after treatment (*n* = 7 animals per group). (**b**) Micro-CT images show inflammatory- and fibrosis-phase mouse lungs after bleomycin administration. The graphs show the Hounsfield unit (HU). (**c**) Representative images of the lungs; hematoxylin and eosin and Masson trichrome staining in lung tissues from C57BL/6 mice 2 weeks after irradiation with or without Nin, PLX, and a combination of Nin and PLX; scale bar = 100 µm. The fibrosis grade score is shown as an Ashcroft score graph. Collagen deposits were quantified using more than five fields (magnification: 200×). For the Ashcroft score graph, error bars indicate SD. For the other graphs, the error bars indicate SEM. ** *p* < 0.01, *** *p* < 0.001, and **** *p* < 0.0001 (two-way ANOVA for multiple comparisons).

Recent studies have suggested that the modulation of macrophage plasticity facilitates the regeneration of fibrotic tissue. Although the survival rate of C57BL6 mice following bleomycin administration remained unchanged in the nintedanib and PLX3397 monotherapy groups, the survival rate increased significantly in the nintedanib and PLX3397 combined treatment groups (Figure 5a). Therefore, following treatment with bleomycin, we administered the drugs (daily) orally for two weeks and conducted micro-CT analysis after 14 days to confirm the induction of PF in the subjects, as indicated by the circles (Figure 5b). PF lesions, as indicated by the progressive greyscale due to bleomycin administration, were most effectively reduced by combined treatment with nintedanib and PLX3397. Furthermore, the fibrotic process and collagen composition decreased synergistically compared to those in the groups treated with nintedanib and PLX3397 alone (Figure 5c). These results indicated that the therapeutic effect of nintedanib on bleomycin-induced fibrosis was significantly enhanced by co-treatment with PLX3397. This combination effectively reduced the mortality due to bleomycin-induced fibrosis, suggesting that it is a potential therapeutic approach for IPF.

### 2.5. Effects of M1/M2 Macrophage Polarization on Vascularization During BIPF

**Figure 6:**
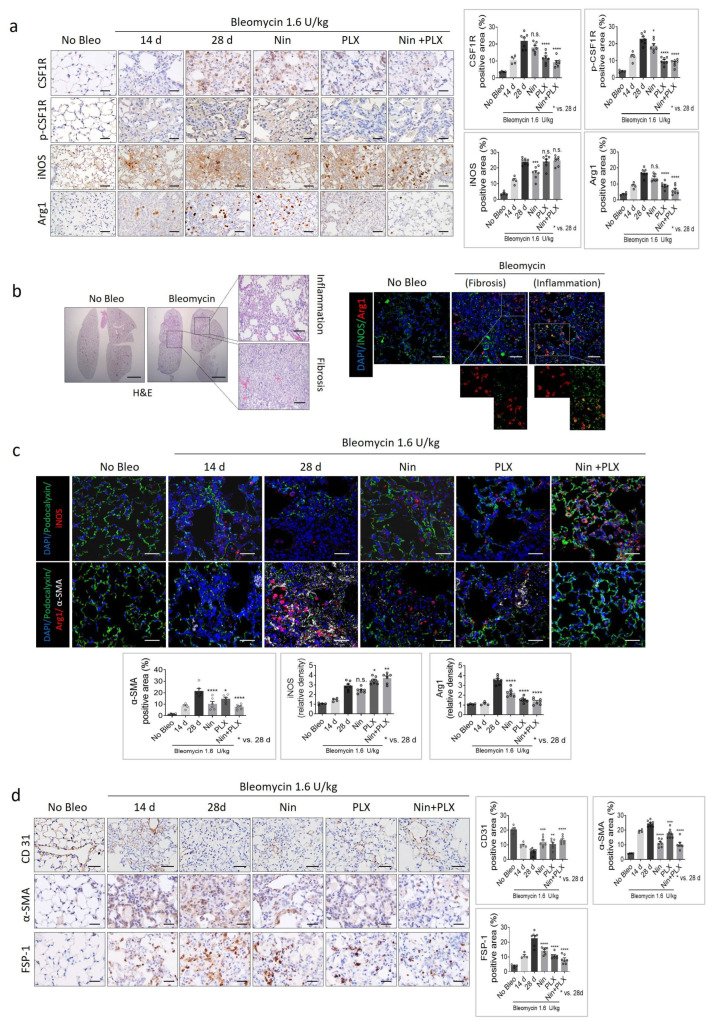
The effects of M1/M2 macrophage polarization on vascularization during BIF. Mice were injected intratracheally with bleomycin (1.6 U/kg) in 60 μL PBS. Mice were orally administered 60 mg/kg of Nin, PLX, or a combination of Nin and PLX 14 days after bleomycin administration. (**a**) Immunohistochemical detection of CSF1R, p-CSF1R, iNOS, and Arg1 in lung tissues obtained from C57BL/6 mice 4 weeks after bleomycin injection with or without Nin, PLX, and combination of Nin and PLX (*n* = 7 animals per group). Scale bar = 20 µm. The average of five fields of CSF1R, p-CSF1R, iNOS, and Arg1 density was quantified (magnification: 200×). (**b**) Representative images of the 10× lungs; hematoxylin and eosin (H&E) in the lung tissues from C57BL/6 mice with or without bleomycin injection (scale bar = 50 µm). Immunofluorescence staining of DAPI (blue), Arg1 (red) and iNOS (green) in lung tissues from C57BL/6 mice 4 weeks after bleomycin injection. (**c**) Immunofluorescence staining of Podocalyxin (green), Arg1 (red), iNOS (red), DAPI (blue) and α-SMA (white). (scale bar = 20 µm). The graph shows the percentage of α-SMA+ area and relative iNOS and Arg1 density. (**d**) Immunohistochemical detection of CD31, α-SMA, and FSP-1 in lung tissues. scale bar = 20 µm. The graphs show the positive area of CD31, α-SMA and FSP-1. The average of five fields of CD31, α-SMA, and FSP-1 density was quantified (magnification: 200×). For graphs, error bars indicate SEM. Each circle in the bar graph represents the mean value of an individual. * *p* < 0.05, ** *p* < 0.01, *** *p* < 0.001, **** *p* < 0.0001, and ns: not significant (one-way ANOVA for multiple comparisons).

Similarly to RIPF, the effect of co-treatment on M2 macrophage polarization and pulmonary vasculature was determined. In the experimental group (Figure 6a), it was observed that 14 days after bleomycin injection, the fibrotic tissue exhibited an accumulation of the Arg1-positive M2 population. Conversely, in the vehicle-treated group, two weeks post-treatment, an increase in CSF1R expression was noted along with the accumulation of the Arg1 macrophage population exceeding, indicating a severe fibrosis state. Notably, in the PLX3397-treated group, there was a significant reduction in the CSF1R expression, coupled with a marked decrease in the Arg1-positive M2 population and, conversely, an increase in M1 macrophages around the inflammatory area. These phenomena were more pronounced with the combined treatment of nintedanib and PLX3397 (Figure 6a,b).

iNOS and Arg 1 co-staining revealed the presence of Arg 1-positive macrophages within the fibrotic area, whereas iNOS^+^ Arg 1^+^ macrophages were observed in the surrounding inflammatory region. This suggests a transition from M1 macrophages to M2 within the fibrotic region (Figure 6b). However, in the PLX3397 treatment group, there was a marked decrease in the Arg1^+^ population, and in the combination therapy group, the Arg1 population was barely observable, predominantly comprising the iNOS^+^ population (Figure 6c). Furthermore, consistent with this shift in the M1/M2 population, the damaged alveolar structure and vascular destruction in BIPF were repaired by the combined therapy.

IHC analysis by CD31 staining revealed recovery of decreased CD31-positive capillary vessels in the fibrotic tissue following combined therapy. Additionally, the area positive for α-SMA and the population of FSP-1-positive cells were significantly reduced (Figure 6d).

Based on these results, we propose that combined treatment with PLX3397 and nintedanib can effectively regulate M2-M1 macrophage polarization, thereby controlling the vascularization of fibrotic tissue and enhancing the therapeutic efficacy of BIF treatment.

### 2.6. Cell-Level Characteristics of Patients with IPF Analyzed Using Single-Cell RNA Sequencing Data

**Figure 7:**
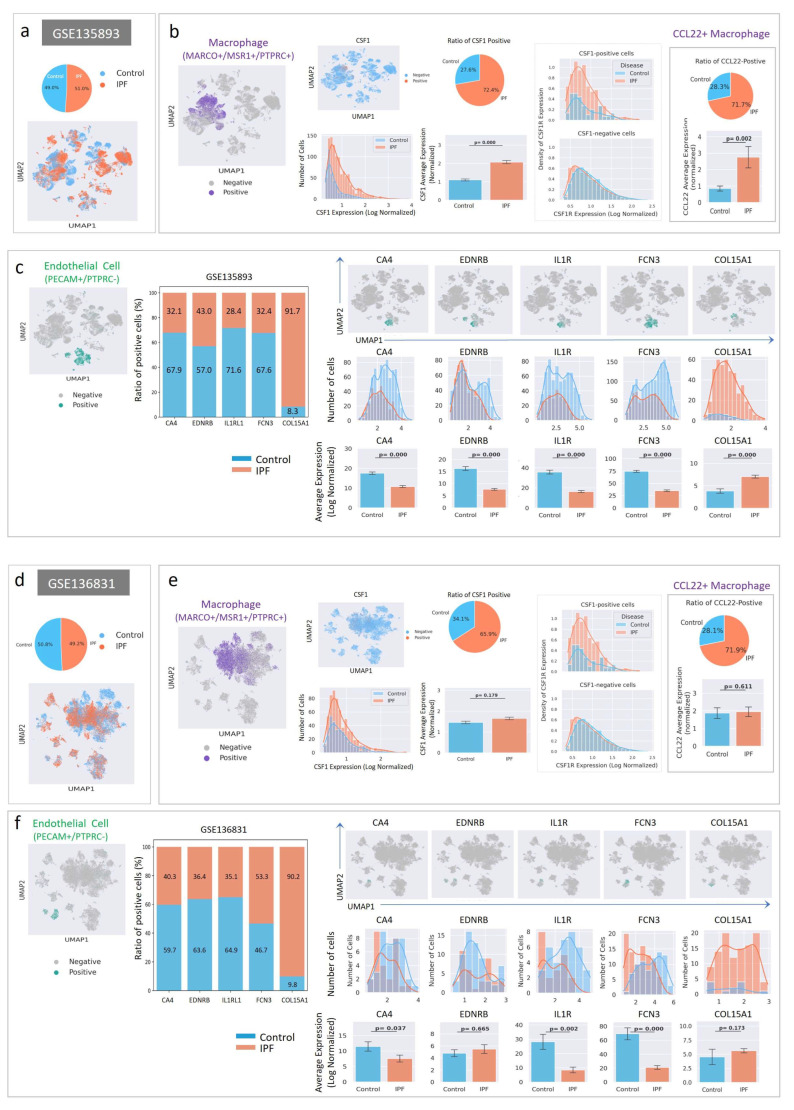
Single-cell RNA sequencing data analysis of patients with IPF. Single-cell RNA sequencing data from patients with IPF (GSE135893: (**a**–**c**), GSE136831: (**d**–**f**)) were downloaded from the Gene Expression Omnibus (GEO) public repository. (**a**,**d**) Both categories of cells (control and IPF) were randomly selected, and the cell numbers were aligned similarly. The ratios of the two categories are also presented. The data were processed using SCANPY (version 1.9.8) and single-cell variational inference (scVI, version 0.14.6) Python packages. Processed data were visualized using the UMAP plotting function. (**b**,**e**) The indicated cell types were assigned with markers specific for macrophages (MARCO^+^/MSR1^+^/PTPRC^+^) and (**c**,**f**) endothelial cells (PECAM1^+^/PTPRC^−^). The assigned cell types were visualized using the UMAP plotting function. CSF1-positive macrophages were analyzed and shown in various plots using the SCANPY and seaborn packages (version 0.12.2). CSF1R, a receptor for CSF1, was compared between CSF1-positive and negative macrophages. (**b**,**e**) CCL22-positive macrophages were analyzed for macrophage subtypes. (**c**,**f**) For capillary endothelial cells, CA4-, EDNRB-, IL1RL1-, and FCN3-positive endothelial cells were analyzed. For peri bronchial endothelial cells, COL15A1-positive endothelial cells were analyzed. The error bars indicate the standard error. *p*-values (Mann–Whitney U test) are shown in the bar graph and relative iNOS and Arg1 density.

CSF1/CSF1R signaling may play a crucial role in both the onset and development of IPF. To acquire insight into these processes, two independent scRNA sequencing datasets from patients with IPF were analyzed (Figure 7). The Gene Expression Omnibus (GEO) series GSE135893 contains experimental data from 12 IPF and 10 control lungs [28], whereas the GEO series GSE136831 contains data from 32 IPF and 28 control lungs. To facilitate downstream analysis, cells from each category (IPF vs. control) were randomly selected, and the cell numbers were aligned (Figure 7a,d). The data from each group was subjected to the same procedure, including filtering, integration, normalization, and dimensionality reduction (Figure 7a,d). Then, cell types were assigned using specific markers: PTPRC^+^/MSR1^+^/MARCO^+^ cells were assigned as macrophages, and PECAM^+^/PTPRC^−^cells were assigned as ECs. Orange dots in the Uniform Manifold Approximation and Projection (UMAP) plot indicate cells expressing the set of markers (Figure 7b,c,e,f).

Since CSF1 is important for macrophage polarization [17], we compared the expression patterns of CSF1 in IPF and control macrophages. CSF1-positive macrophages were selected and shown in the UMAP plot (Figure 7b,e), indicating that CSF1 was expressed in a subset of macrophages. The number of CSF1-positive macrophages and macrophagic CSF1 expression (Figure 7b,e) were higher in IPF lungs than in the controls. However, the increase in CSF1 expression in GSE136831 was not statistically significant. In addition, co-expression of CSF1 and its receptor (CSF1R) was more frequent in IPF macrophages, suggesting a possible feed-forward activation of CSF1/CSF1R signaling.

Macrophage population analyses using the two datasets did not yield consistent results. However, the number of Cys-Cys Motif Chemokine Ligand 22 (CCL22)-positive macrophages and the expression level of CCL22 increased in IPF (Figure 7b,e; Right panel), suggesting that CCL22-producing macrophages may control the immunological response [29].

Our data showed that vessel formation was greatly affected by the targeting of CSF1R in bleomycin and RIPF mouse models (Figure 4c and Figure 6c). Since CSF1/CSF1R signaling was upregulated in IPF lungs, we investigated the possible changes in endothelial populations in IPF lungs. To detect changes, capillary EC markers, such as carbonic anhydrase 4 (CA4), endothelin receptor subtype B (EDNRB), interleukin-1 receptor-like 1 (IL1RL1), and ficolin-3 (FCN3), and parabronchial endothelial markers, collagen type XV alpha 1 (COL15A1), were measured in PECAM1^+^/PTPRC^−^ECs. ECs expressing these genes are shown in the UMAP plot (Figure 7c,f). The number of ECs expressing capillary markers was decreased in patients with IPF, and the level of capillary marker expression in ECs was downregulated. In contrast, the number of COL15A1-positive ECs and the level of endothelial COL15A1 expression increased in IPF lungs.

## 3. Discussion

In this study, we aimed to enhance the efficacy of nintedanib by potentially amplifying its therapeutic effects on both RIPF and BIPF and potentially increasing the survival rate of BIF in vivo via combination treatment (nintedanib and PLX3397). Thus, we investigated macrophage polarization in an RIPF animal model. Compared with the heterogeneity of fibrotic tissue in the BIPF model, this model offers the advantage of easily observing the accumulation of macrophages within the tissue from the inflammatory stage induced by radiation to fibrosis development. We observed that only Arg1^+^ M2 macrophages accumulated in severely fibrotic areas of RIPF. In the area surrounding the focal irradiation, where radiation exposure was comparatively weak, M1 macrophages were predominantly observed, a pattern similar to that observed in BIPF tissues. Notably, iNOS^+^ Arg1^+^ macrophages were observed at the interface between the regions where M1 and M2 macrophages were observed, indicating the possibility of trans-differentiation between the two macrophage subtypes. In addition to its function as an inhibitor of PDGFR, FGFR, and VEGFR, nintedanib targets CSF1R [30]. CSF1R targeting in macrophages via nintedanib enhances IL-4 signaling, resulting in antifibrotic effects. In this study, we observed a decrease in the M2 macrophage population in tissues treated with nintedanib, demonstrating reduced fibrosis in the RIPF and BIF models.

However, in the PLX3397-treated group, a reduction in the M2 macrophage population and a simultaneous increase in the M1 macrophage population were observed, which appeared to correlate with the extent of fibrosis inhibition in the RIPF and BIF models. Interestingly, when the two drugs were combined, the M2 macrophage population decreased, while the proportion of M1 macrophages increased significantly. Although M1 macrophages are typically pro-inflammatory, their increase after Pexidartinib and Nintedanib combination therapy may be beneficial. This shift reflects a reduction in profibrotic M2 macrophages, which promote fibrosis through the TGF-β/Smad pathway [31]. M1 macrophages, in the resolution phase of fibrosis, may contribute to ECM clearance, inhibit myofibroblast activation, and restore immune balance [14,19]. Therefore, the observed increase in M1 macrophages may support anti-fibrotic remodeling. Because M2-polarized macrophages play a substantial role in advancing both fibrosis and cancer, shifting their polarization to an M1 phenotype or regenerative state has emerged as a promising therapeutic approach. Several studies have shown its effectiveness in experimental cancer treatments [27,28,29].

In vivo investigations have shown that CSF1R inhibition leads to a reduction in M2 macrophages and their repolarization toward the M1 phenotype, resulting in antitumor effects [32]. In this study, the notable changes in the M1/M2 ratio observed after combined treatment with nintedanib and PLX3397 suggested a regenerative effect during the fibrotic phase. We used CT to monitor the therapeutic effects of bleomycin-induced fibrosis in individual mice. The combined treatment restored the established PF with changes in M1/M2 macrophage polarization in tissue fibrosis, demonstrating the regenerative efficacy of combined therapy in tissue fibrosis.

Furthermore, we observed that M1 and M2 macrophages directly influenced the vasculature of ECs. Treatment with nintedanib and PLX3397 in PF reduced the proportion of M2 macrophages while simultaneously increasing the proportion of M1 macrophages, thus preserving the vasculature and maintaining the alveolar structure. Moreover, dysfunction in the functional and structural aspects of blood vessels at the advanced stages of fibrosis is a prominent feature [31,32,33]. However, studies on the regulation of vasculature in PF remain limited.

A dysfunctional vasculature is a hallmark of advanced fibrosis. Recent evidence suggests that increased vascular permeability in IPF, with EC-like myofibroblasts associated with abnormal vasculature, contributes to fibrosis progression. Persistent permeability and vascular leakage in the lungs may establish and exacerbate a profibrotic environment [33]. Previous studies have indicated that nintedanib leads to a significant improvement in pulmonary microvascular architecture and reduces vascular proliferation [34].

The insights gained from single-cell RNA sequencing data of patients with IPF provide valuable information regarding the dysregulation of macrophage polarization and ECs, shedding light on the potential therapeutic targets for further investigation. CSF1 upregulation in macrophages observed in patients with IPF suggests an enhanced activation of macrophages, potentially contributing to the fibrotic process. These findings underscore the importance of targeting macrophage polarization as a therapeutic strategy for IPF management. Furthermore, the downregulation of capillary EC markers, such as CA4, EDNRB, IL1RL1, and FCN3, indicates capillary vessel dysfunction in patients with IPF. This dysfunctional vascular phenotype may contribute to the progression of fibrosis by impairing tissue perfusion and oxygenation [35]. The significantly increased expression of parabronchial endothelial markers, specifically COL15A1, suggests the presence of abnormal peri bronchial foci during PF development of PF. Abnormalities in the peri bronchial vasculature may exacerbate fibrotic processes by promoting inflammation and tissue remodeling.

In conclusion, combination therapy with PLX3397 and nintedanib represents a promising approach for the treatment of IPF by targeting macrophage polarization and enhancing the normalization of impaired microvascular architecture. Further research is required to identify other effective therapeutic interventions and to translate them into clinical practice.

## 4. Materials and Methods

### 4.1. Mice and Ethical Approval

All animal experiments were reported in accordance with the ARRIVE guidelines. Mice experiments were approved by the Institutional Animal Care and Use Committee of the Korea Institute of Radiological & Medical Sciences (Kirams 2020-0014, 2021-0093). Male mice (C57BL/6) aged 6–8 weeks were obtained from Orient Bio, Co., Ltd, Seongnam, Gyeonggi, Republic of Korea. Specific pathogen-free C57BL/6 Lyz2-Cre, CAG-tdTomato mice were purchased from the Jackson Laboratory, Bar Harbor, ME, USA. Lyz2-Cre; tdTomato mice were generated by crossing Lyz2-Cre mice with CAG-tdTomato mice. All animal experimental data provided are representative of three independent experiments. Animals were randomly assigned to control and treatment groups, and treatments and measurements were performed in a randomized order to minimize potential confounders. All experiments were conducted with 6–8-week-old mice, and the mice were maintained on a 12 h light-dark cycle in a standard environment (20 ± 1 °C room temperature, 50 ± 10% relative humidity) with a standard diet and water ad libitum. All mice were anesthetized with a combination of anesthetics before being euthanized.

### 4.2. RIPF Model

Radiation exposure was performed using the X-RAD 320 system (Precision X-Ray, Inc., Madison, CT, USA), as previously described [36]. Briefly, the left main bronchi of 7- to 8-week-old C57BL/6 mice were exposed to a radiation dose of 90 Gy using a 4 mm diameter field. One hour before irradiation, nintedanib (60 mg/kg), PLX3397 (60 mg/kg), or a combination of nintedanib and PLX3397 (60 mg/kg) was administered orally. Subsequently, treatment administration was continued once daily for 13 days.

### 4.3. Bleomycin-Induced PF (BIPF Model)

Six-week-old male C57BL/6 mice were obtained from Orient Bio, Co., Ltd, Seongnam, Gyeonggi, Republic of Korea. Intratracheal injection of bleomycin (1.6 U/kg; Sigma-Aldrich, St. Louis, MO, USA; #B5507) dissolved in 60 μL PBS was administered orally to the mice [37]. Oral administration of 60 mg/kg nintedanib, 60 mg/kg PLX3397, or a combination of nintedanib and PLX3397 (60 mg/kg) was initiated 14 days after bleomycin administration. The drugs were orally administered daily for 2 weeks.

### 4.4. Immunohistochemistry and Immunofluorescence Staining

The tissues were immersed in a solution of 10% neutral buffered formalin for fixation, followed by embedding in paraffin, and then sectioned into tissue slices. Following deparaffinization, tissue slides were stained with H&E. The fibrosis grade was evaluated according to the Ashcroft score [38]. Collagen deposition was evaluated through Masson trichrome staining using the Polyscience staining kit (Polysciences, Inc., Warrington, PA, USA; #25088-100). For IHC staining, deparaffinized sections underwent antigen retrieval in citrate buffer at 95 °C for 30 min, followed by treatment with 0.3% hydrogen peroxide in methanol for 15 min. The tissue slides were permeabilized for 15 min using PBS containing 0.1% Triton X-100, followed by blocking in PBS containing normal horse serum at room temperature for 30 min. For fluorescence staining, 0.3% hydrogen peroxide treatment was omitted. Primary antibody incubation was performed overnight at 4 °C in a blocking buffer. For immunohistochemical staining, after the secondary antibody reaction, a brown color was developed using ABC (#SK-6100) and DAP (#SK-4100) kits (Vector Laboratories, Inc., Burlingame, CA, USA), according to the manufacturers’ instructions, and the nucleus was stained with hematoxylin. Immunofluorescence staining was performed using a fluorescent secondary antibody, and the nuclei were stained with DAPI. Quantification involved capturing a minimum of 5 images per section, and the positively stained areas were quantified using ImageJ software 2.16.0v. (http://imagej.net/).

### 4.5. Microcone Beam CT (Micro-CBCT)

The micro-CBCT utilized the following settings: 40 kV and 3 mA with a small focal size, along with an Al filter that allowed adjustment of the tube voltage and X-ray tube settings. The system employed an amorphous silicon flat-panel detector manufactured by Perkin-Elmer in Wiesbaden, Germany, with a pixel size of 200 μm.

### 4.6. Cell Culture and Tube Formation Assay

Human umbilical vein ECs (HUVEC) were obtained from PromoCell (GmbH, Heidelberg, Germany; #C-12203) and cultured in Endothelial Cell Growth Medium 2 (PromoCell, #C-22011) at 37 °C and 5% CO_2_. Cells were passaged up to seven times. HUVECs were exposed to gamma radiation at a rate of 3.81 Gy/min using a 137Cs source (Atomic Energy of Canada, Chalk River, ON, Canada). For tube formation experiments, Human CD14+ Monocytes were obtained from PromoCell (#C12909). Human CD14+ Monocytes were differentiated into macrophages by treatment with GM-CSF (Promocell, #C12914), and M-CSF (Promocell, #C12915) was administered every other day for a period of 7 days [39]. To assess the incorporation of macrophages into capillaries, HUVECs were exposed to radiation and subsequently seeded onto Matrigel (Corning, Corning City, NY, USA, #354230) with or without differentiated macrophages. Analysis was conducted 10 h after seeding, following 72 h of radiation exposure. Before seeding, the macrophages were fluorescently labeled with Vybrant™ Multicolor Cell-Labeling Kit (DiO, 1:500; Invitrogen^TM^, Waltham, MA, USA; #V22889), following the manufacturer’s instructions. THP-1 cells were treated with PMA for 6 h, followed by exposure to LPS (10 ng/mL) and IFNγ (5 ng/mL) for 18 h to induce differentiation into M1 macrophages. Furthermore, to prompt differentiation into M2 macrophages, the cells were exposed to IL-10 (25 ng/mL) and IL-4 (25 ng/mL) for a duration of 18 h.

### 4.7. Single-Cell RNA Sequencing Data Analysis

Two independent single-cell RNA sequencing datasets of patients with IPF were selected for the meta-analysis [28,40]. The GSE135893 and GSE136831 datasets were downloaded from the Gene Expression Omnibus (GEO) public repository. The downloaded data were read and transformed into annotated data (AnnData Python package version 0.10.5, https://doi.org/10.1101/2021.12.16.473007). To facilitate downstream analysis, cells (control vs. IPF) were randomly selected, and the cell numbers were aligned. Data were processed using the standard procedure recommended by ScanPy, a scalable toolkit for analyzing single-cell gene expression data (version 1.9.8) [41]. After cell filtering, latent space embeddings, obtained using single-cell variational inference (scVI, version 0.14.6), were used to minimize batch effects. Subsequently, data from each group were subjected to the same standard normalization, dimensionality reduction, and cell type assignment. Markers specific for macrophages (MARCO^+^/MSR1^+^/PTPRC^+^) and ECs (PECAM1^+^/PTPRC^−^) were used to assign the respective cell types. SCANPY core plotting functions and the Seaborn package (https://doi.org/10.21105/joss.03021, version 0.12.2) were used for graphical representations. Gene expression counts were not normally distributed; therefore, non-parametric statistical comparisons using the Mann–Whitney U test were performed. CCL22 for the macrophage subtype, CA4, EDNRB, IL1RL1, and FCN3 (PanglaoDB (https://panglaodb.se)), capillary ECs, COL15A1 [40], and peri bronchial ECs were used for subtype analysis.

### 4.8. Antibodies for Immunofluorescence and Immunohistochemistry Staining

IHC, and immunofluorescence (IF) staining were performed using primary antibodies against the Argnase-1 (IHC/IF, 1:2000; Novusbio, Centennial, CO, USA; #NBP1-32731), iNOS (IHC/IF, 1:100; Santa Cruz Biotechnology, Dallas, TX, USA; #sc7271), p-CSF1R (IHC, 1:100; Cell Signaling, Danvers, MA, USA; #3155), CSF1R (IHC/IF, 1:100; Santa Cruz Biotechnology; #sc-692), tdTomato (IF, 1:10,000; Lsbio, Seattle, WA, USA; #LS-C340696), CD206 (IF, 1:200; Abcam, Cambridge, UK; #ab64693), CD31 (IHC, 1:200; Abcam; #ab56299), α-SMA (IHC/IF, 1:200; Abcam; #5694) FSP-1 (IF, 1:200; Millipore, Billerica, MA, USA; #ABF-32), Podocalyxin (IF, 1:200; R&D system, Minneapolis, MN, USA; #AF1556). To stain actin stress fibers in cells, they were stained with Alexa Fluor 546-conjugated phalloidin (1:200; Invitrogen; #A22283), which specifically binds to F-actin.

### 4.9. Statistical Analysis

Student’s *t*-test and one-way analysis of variance (ANOVA) were used to explore the statistical significance of differences between experimental groups. All statistical analyses were performed using GraphPad Prism version 5.0 (GraphPad Software Inc., San Diego, CA, USA). Statistical significance was set at *p* < 0.05. The experimenters were blinded to the group assignments and outcome assessments.

## Data Availability

The data utilized and/or produced during this study can be requested from the corresponding author upon reasonable request.
